# Supplementary material for: Risk Factors for Antimicrobial Resistance in Turkey Farms: A Cross-Sectional Study in Three European Countries
Source: Antibiotics (Basel). 2021 Jul 6;10(7):820. doi: 10.3390/antibiotics10070820 (PMC8300668; doi:10.3390/antibiotics10070820)
Supplement: Supplementary file 1 [file antibiotics-10-00820-s001.zip › antibiotics-1258724-supplementary.pdf]

Supplementary Materials

Risk Factors for Antimicrobial Resistance in Turkey Farms: A Cross-Sectional Study in Three European Countries

Mayu Horie [1,*], Dongsheng Yang [1], Philip Joosten [2], Patrick Munk [3], Katharina Wadepohl [4], Claire Chauvin [5], Gabriel Moyano [6], Magdalena Skarżyńska [7], Jeroen Dewulf [2], Frank M. Aarestrup [3], Thomas Blaha [4], Pascal Sanders [5], Bruno Gonzalez-Zorn [6], Dariusz Wasyl [7], Jaap A. Wagenaar [8,9], Dick Heederik [1], Dik Mevius [8,9], Heike Schmitt [1,10], Lidwien A.M. Smit [1] and Liese Van Gompel [1] on behalf of the EFFORT-group[†]

1 Institute for Risk Assessment Sciences, Utrecht University, Yalelaan 2, 3584 CM Utrecht, The Netherlands; d.yang@uu.nl (D.Y.); d.heederik@uu.nl (D.H.); h.schmitt@uu.nl (H.S.); l.a.smit@uu.nl (L.A.M.S.); l.vangompel@uu.nl (L.V.G.)
2 Veterinary Epidemiology Unit, Department of Obstetrics, Reproduction and Herd Health, Faculty of Veterinary Medicine, Ghent University, Salisburylaan 133, 9820 Merelbeke, Belgium; philip.joosten@ugent.be (P.J.); jeroen.dewulf@ugent.be (J.D.)
3 Research Group for Genomic Epidemiology, The National Food Institute, Technical University of Denmark, Kemitorvet, 2800 Kgs. Lyngby, Denmark; pmun@food.dtu.dk (P.M.); fmaa@food.dtu.dk (F.M.A.)
4 Field Station for Epidemiology, University of Veterinary Medicine Hannover, Büscheler Straße 9, 49456 Bakum, Germany; katharina.wadepohl@tiho-hannover.de (K.W.); thomas.blaha@tiho-hannover.de (T.B.)
5 Epidemiology, Health and Welfare Unit, The French Agency for Food, Environmental and Occupational Health & Safety (ANSES), 22440 Ploufragan, France; claire.chauvin@anses.fr (C.C.); pascal.sanders@anses.fr (P.S.)
6 Antimicrobial Resistance Unit (ARU), Animal Health Departement, Faculty of Veterinary Medicine and VISAVET Health Surveillance Centre, Complutense University of Madrid, 28040 Madrid, Spain; gmoyano@ucm.es (G.M.); bgzorn@ucm.es (B.G.-Z.)
7 Department of Microbiology, National Veterinary Research Institute (PIWet), Partyzantów Avenue 57, 24-100 Puławy, Poland; magdalena.skarzynska@piwet.pulawy.pl (M.S.); wasyl@piwet.pulawy.pl (D.W.)
8 Department of Infectious Diseases and Immunology, Faculty of Veterinary Medicine, Utrecht University, Yalelaan 1, 3584 CL Utrecht, The Netherlands; j.wagenaar@uu.nl (J.A.W.); d.j.mevius@uu.nl (D.M.)
9 Department of Bacteriology and Epidemiology, Wageningen Bioveterinary Research, Houtribweg 39, 8221 RA Lelystad, The Netherlands;
10 National Institute for Public Health and the Environment, P.O. Box 1, 3720 BA Bilthoven, The Netherlands

* Correspondence: m.horie@uu.nl
† EFFORT-group: see acknowledgements

Supplementary Materials Part A: Supplementary figures and tables

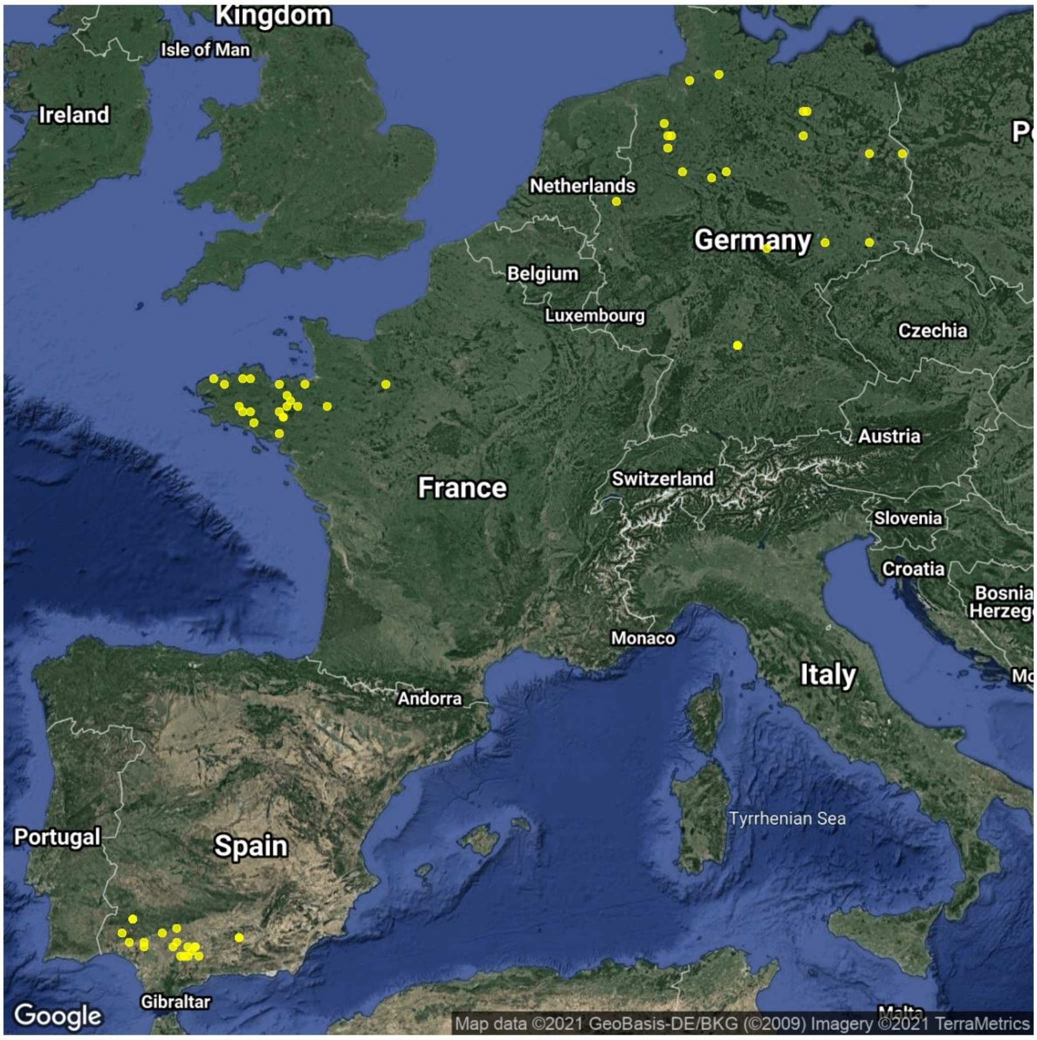

Figure S1. Distribution of the 60 turkey farms across three countries. Yellow dots represent the sampled farms. German farms were geographically spread over the country, while all French and Spanish farms were concentrated in Brittany and Andalusia, respectively, both being the major turkey production sites of these countries.

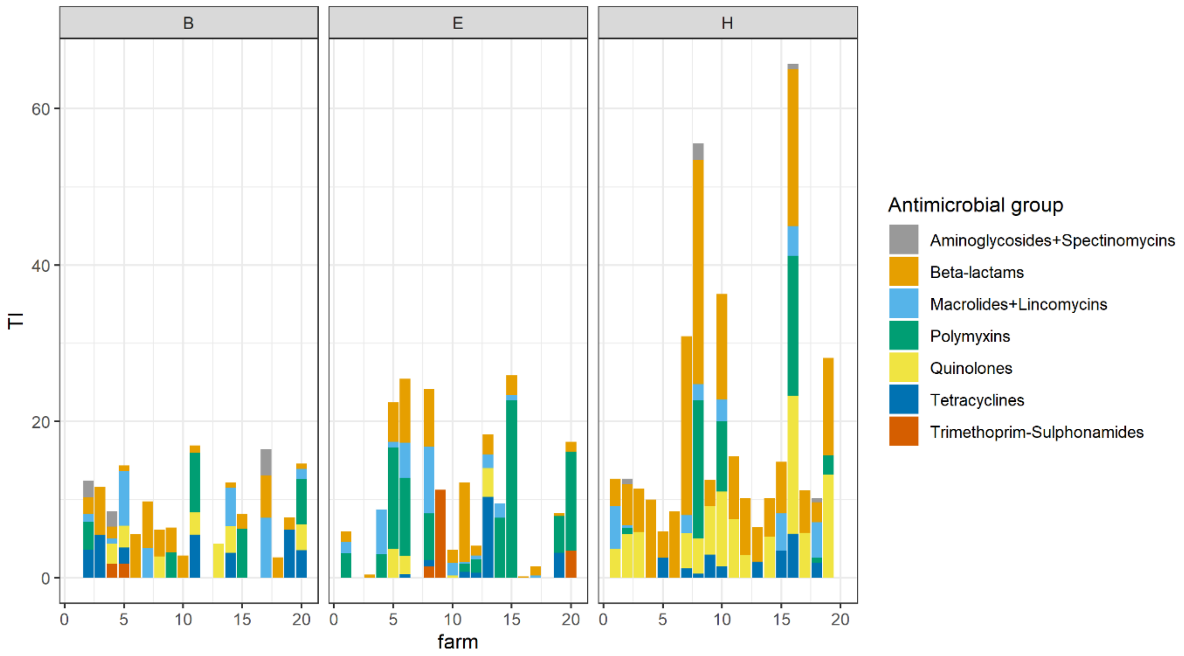

Figure S2. Antimicrobial usage in 60 turkey farms in three countries, expressed as the sum of treatment incidence (TI). TI was calculated for lincomycin-spectinomycin combination product and subsequently divided and added to macrolides and aminoglycosides, respectively. Beta-lactams included aminopenicillins and penicillins. Quinolones included fluoroquinolones and other quinolones (flumequine).in farms per country and per antimicrobial class.

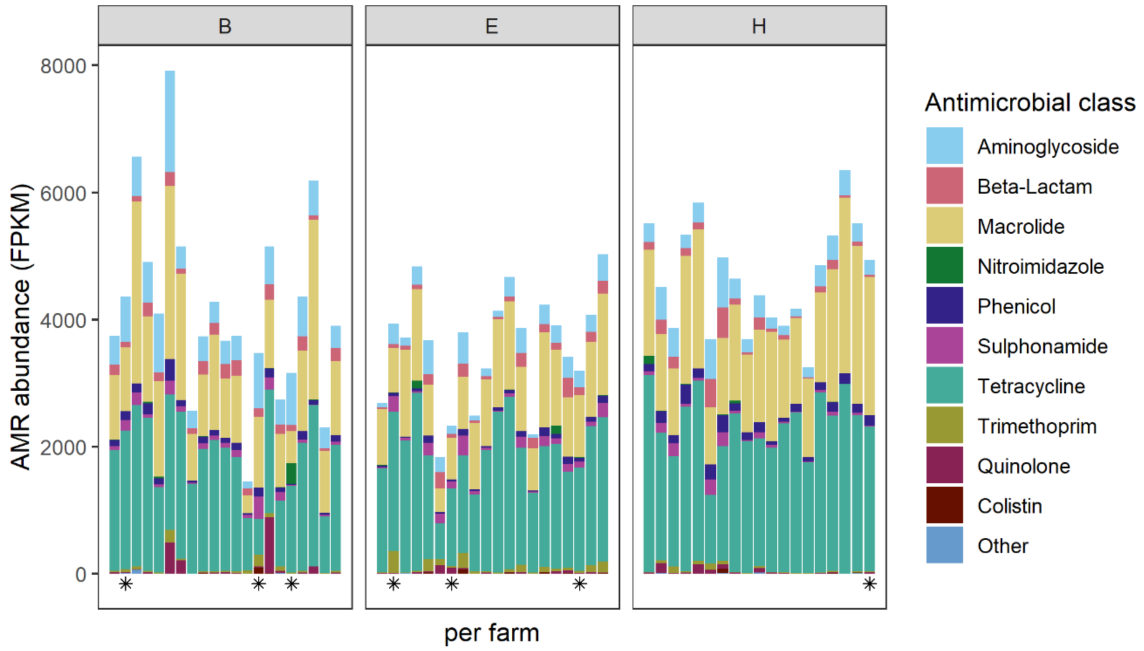

Figure S3. Abundance of antimicrobial resistance (AMR) genes per farm, expressed as fragments per kilobase reference per million bacterial fragments (FPKM). Columns represent 60 samples from three countries (B: $n = 21$, E: $n = 20$, and H: $n = 19$). One additional farm was visited in country B due to incomplete questionnaire data in one of the farms, resulting in twenty-one samples in total. One sample in country H was removed due to errors during processing. Seven farms where no antimicrobial use was reported in the sampled flock are indicated with an asterisk below the columns.

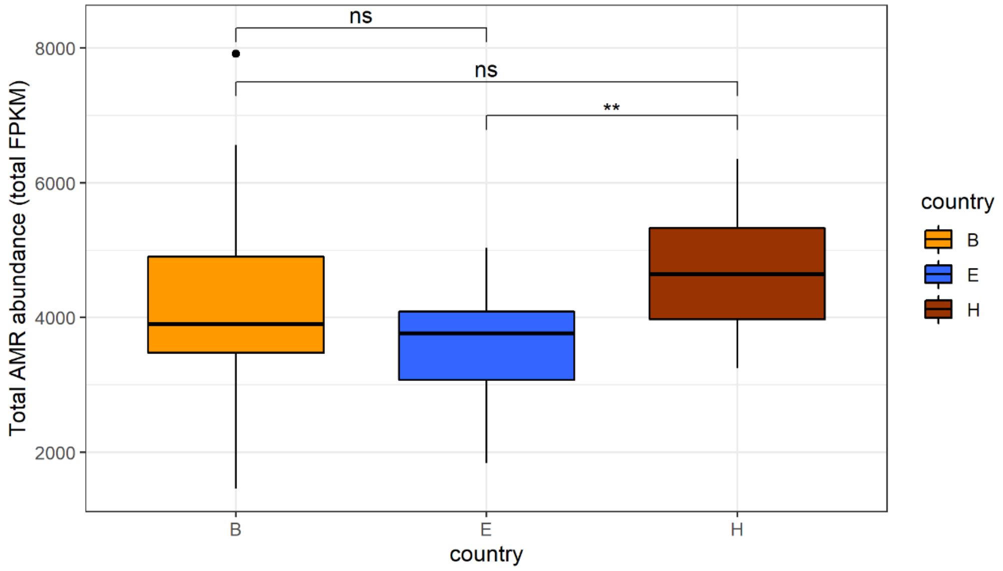

Figure S4. Total abundance of antimicrobial resistance (AMR) genes per country, expressed as the sum of fragments per kilobase reference per million bacterial fragments (FPKM). In total 60 samples from three countries (B: $n = 21$, E: $n = 20$, and H: $n = 19$) were included. One additional farm was visited in country B due to incomplete questionnaire data in one of the farms, resulting in twenty-one samples in total. One sample in country H was removed due to errors during processing. The mean total abundance on the farms in country E was significantly lower than that of country H (by one-way ANOVA and Tukey's HSD). ** p<0.01, ns= not significant.

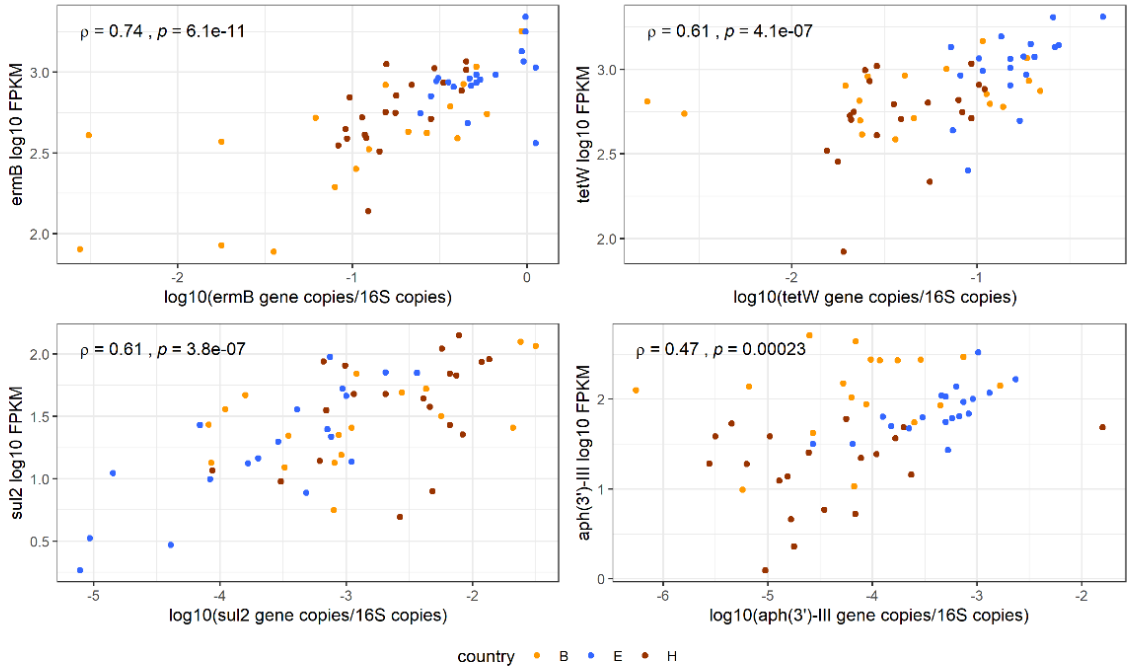

Figure S5. Correlations (Spearman ϱ) between the abundance of *ermB*, *tetW*, *sul2* and *aph3′-III* gene level clusters detected by metagenomics (vertical axis) and the abundance of these genes detected by qPCR (horizontal axis) Dots represent 57 farms (country B: $n = 21$, E: $n = 20$, and H: $n = 19$). Metagenomics samples were pooled at farm level whereas for qPCR, the median of the samples per farm were used. FPKM = fragments per kilobase reference per million bacterial fragments.

Table S1. Univariate associations between antimicrobial usage (AMU), technical farm characteristics, biosecurity measures of the turkey farms and the median relative faecal abundance of *ermB*, *tetW*, *sul2* and *aph3′-III* per farm

**Model variables**	***ermB***		***tetW***		***sul2***		***aph3′-III***	
	GMR	[95%CI]	GMR	[95%CI]	GMR	[95%CI]	GMR	[95%CI]
**AMU**								
Log_{10} TI macrolides + lincomycin	1.72	[0.74,3.98]	1.23	[0.60,2.53]	2.38	[0.60,9.36]	1.82	[0.46,7.17]
Log_{10} TI tetracyclines	1.75	[0.72,4.30]	1.72	[0.80,3.69]	0.79	[0.18,3.46]	1.30	[0.30,5.70]
Trimethoprim-sulphonamides used (ref:no)	**2.74**	**[1.06,7.11]**	1.70	[0.74,3.92]	3.92	[0.80,19.14]	0.31	[0.06,1.50]
Aminoglycosides or spectinomycin used (ref:no)	1.90	[0.81,4.42]	1.62	[0.79,3.36]	0.95	[0.23,3.87]	1.63	[0.40,6.59]
Log_{10} TI total AMU	**1.86**	**[1.03,3.37]**	**1.81**	**[1.10,3.00]**	1.53	[0.56,4.13]	1.05	[0.39,2.84]
**Technical farm characteristics**								
Log_{10} total number of turkeys at the farm	0.45	[0.19,1.08]	0.55	[0.26,1.15]	2.50	[0.75,8.34]	0.85	[0.20,3.62]
Age of turkeys at sampling (standardized)	0.86	[0.60,1.22]	0.88	[0.65,1.19]	1.20	[0.42,3.41]	0.68	[0.39,1.21]
Other livestock present (ref:no)	0.84	[0.46,1.55]	0.87	[0.52,1.47]	1.72	[0.64,4.62]	0.37	[0.14,0.97]
Sampling season (ref: autumn, winter)	1.43	[0.68,2.99]	1.46	[0.78,2.75]	0.34	[0.11,1.06]	0.89	[0.26,3.01]
**Biosecurity**								
Visitor access more than once a month (ref:no)	**0.32**	**[0.17,0.62]**	**0.34**	**[0.20,0.59]**	0.67	[0.21,2.15]	0.45	[0.14,1.41]
Outdoor access possible for turkeys (ref:no)	**0.31**	**[0.13,0.75]**	**0.37**	**[0.17,0.80]**	3.9	[0.86,17.6]	0.43	[0.09,2.00]
Different age categories of turkeys present (ref:no)	**0.38**	**[0.20,0.72]**	**0.47**	**[0.27,0.82]**	0.5	[0.17,1.54]	1.63	[0.53,4.96]
Bird- and vermin proof grids placed on the air inlets (ref:no)	0.95	[0.40,2.29]	1.16	[0.55,2.44]	1.67	[0.40,6.94]	**5.72**	**[1.48,22.1]**
Staff keeps turkeys or birds at home (ref:no)	0.67	[0.31,1.42]	0.69	[0.36,1.32]	2.21	[0.65,7.56]	**0.21**	**[0.07,0.68]**
Disinfecting footbaths present on the farm (ref:no)	0.70	[0.41,1.20]	**0.63**	**[0.40,0.98]**	0.85	[0.35,2.08]	0.66	[0.27,1.60]

Associations in bold have a p value < 0.05. Biosecurity variables displayed in the table are those significantly associated with the abundance of at least one of the gene. AMU=Antimicrobial usage; GMR=Geometric mean ratio; 95%CI=95% Confidence interval; TI=Treatment incidence.

Table S2. Univariate associations between antimicrobial usage (AMU), characteristics and biosecurity measures of the turkey farms and the occurrence of *E. coli* isolates from turkey faeces resistant to ampicillin, tetracycline and ciprofloxacin

Model variables	Values	Susceptible isolates (WT) n (%)	Resistant isolates (NWT) n (%)	Odds Ratio	[95%CI]
**AMP**					
**AMU**					
Log_{10} TI aminopenicillins				**6.08**	**[2.14,17.27]**
Log_{10} TI total AMU				**2.52**	**[1.69,3.78]**
**Technical farm characteristics**					
Log_{10} total number of turkeys at the farm				3.26	[0.76,14.02]
Age of turkeys at sampling (standardized)				**0.62**	**[0.39,0.99]**
Other livestock present (ref: no)	Yes	49 (43)	140 (29)	0.39	[0.14,1.03]
	No	64 (57)	343 (71)		
Sampling season (ref: AW)	SS	44 (61)	242 (50)	1.45	[0.57,3.70]
	AW	69 (39)	241 (50)		
**Biosecurity**					
Other livestock farms present within 500m	Yes	87 (77)	279 (58)	**0.32**	**[0.12,0.86]**
	No	26 (23)	204 (42)		
**TET**					
**AMU**					
Log_{10} TI tetracyclines				**5.30**	**[1.03,27.12]**
Log_{10} TI total AMU				**2.36**	**[1.61,3.46]**
**Technical farm characteristics**					
Log_{10} total number of turkeys at the farm				1.32	[0.29,6.04]
Age of turkeys at sampling (standardized)				**0.62**	**[0.38,1.00]**
Other livestock present (ref: no)	Yes	46 (38)	143 (30)	0.62	[0.22,1.75]
	No	74 (62)	333 (70)		
Sampling season (ref: AW)	SS	29 (24)	257 (54)	**4.64**	**[1.86,11.57]**
	AW	91 (76)	219 (46)		
**Biosecurity**					
Wild birds can enter the stables (ref: no)	Yes	10 (8)	139 (29)	**5.42**	**[1.67,17.63]**
	No	110 (92)	337 (71)		
Different age categories of turkeys present (ref: no)	Yes	51 (43)	99 (21)	**0.23**	**[0.08,0.64]**
	No	69 (58)	377 (79)		
CIP					
**AMU**					
Log_{10} TI quinolones				**11.09**	**[3.25,37.83]**

Log$_{10}$ TI total AMU				**1.86**	**[1.27,2.71]**
**Technical farm characteristics**					
Log$_{10}$ total number of turkeys at the farm				0.85	[0.20,3.65]
Age of turkeys at sampling (standardized)				0.90	[0.50,1.61]
Other livestock present (ref: no)	Yes	46 (38)	143 (30)	0.63	[0.24,1.69]
	No	74 (62)	333 (70)		
Sampling season (ref: AW)	SS	29 (24)	257 (54)	1.01	[0.27,3.80]
	AW	91 (76)	219 (46)		
**Biosecurity**					
The nearest turkey farm within 500m (ref: no)	Yes	88 (27)	41 (15)	**0.34**	**[0.12,0.96]**
	No	234 (73)	233 (85)		

Associations in bold have a p value < 0.05. Biosecurity variables displayed in the table are those significantly associated with AMR. AMU=Antimicrobial usage; WT= wild type; NWT= non-wild type; 95%CI=95% Confidence interval; AMP=ampicillin; TET=tetracycline; CIP=ciprofloxacin; TI=Treatment incidence; SS= Spring and Summer; AW= Autumn and Winter.

Table S3. Ten most abundant antimicrobial resistance (AMR) genes in turkey faeces quantified by metagenomics and their proportion within the macrolide, tetracycline, sulphonamide and aminoglycoside class clusters

	Macrolide	%	Tetracycline	%	Sulphonamide	%	Aminoglycoside	%
1	***erm(B)*_clust**	**69.0**	***tet(W)***	**42.3**	***sul2***	**42.6**	***aph(3')-III***	**25.3**
2	*mef(A)*-3	5.5	*tet(L)*_clust1	13.8	*sul1*	36.4	*aadA_ant(3'')-Ia*_clust	14.9
3	*mdf(A)*	4.4	*tet(M)*	13.5	*sul3*_2_AJ459418	21.0	*ant(6)-Ia*_clust1	12.1
4	*erm(F)*_clust	4.0	*tet(Q)*	8.6			*aph(3'')-Ib*	8.0
5	*erm(C)*_clust	3.8	*tet(A)*	4.9			*aph(6)-Id*	8.0
6	*mef(A)*_clust	3.6	*tet(O/W/32/O/W/O)*	3.1			*aadA*_clust1	7.9
7	*erm(T)*_clust	2.3	*tet(40)*	2.8			*ant(6)-Ia*_clust2	7.3
8	*msr(D)*	1.3	*tet(44)*	1.9			*aadD*	3.5
9	*erm(X)*_clust	1.3	*tet(Z)*	1.5			*ant(9)-Ia*-1	2.8
10	*erm(T)*_4_AJ488494	1.1	*tet(X)*_clust	1.4			*aph(3')-Ia_aph(3')-Ic*	2.3

Fragments per kilobase reference per million bacterial fragments (FPKM) values of AMR genes were clustered at the 90% identity level. Genes in bold were used as qPCR target. Only three gene-level clusters were found for sulphonamide.

Supplementary Materials Part B: Selected biosecurity check questions from the questionnaire used in risk factor analyses. Answers are shown in brackets with the number of farms.

- Is the farm site divided into a clean and dirty area?

  ☐ Yes (9)

  ☐ No (51)

- Are visitors obliged to check in before having entrance to the stables?

  ☐ Yes (47)

  ☐ No (13)

- How many times a month is access to the stables granted for visitors (family members, technicians, representatives, etc.)?

  ☐ Never (16)

  ☐ 1-12 times (27)

  ☐ > 12 times (17)

- Is there permanent staff that also keeps turkeys or birds at home?

  ☐ Yes (10)

  ☐ No (50)

- Is there permanent staff that also works on other turkey farms?

  ☐ Yes (6)

  ☐ No (54)

- Is equipment present on the farm that is also used on other farms (for example, a straw blower)?

  ☐ Yes (14)

  ☐ No (46)

- Is outdoor access possible for the turkeys?

  ☐ Yes (14)

  ☐ No (46)

- Can wild birds enter the stable(s)?

☐ Yes (15)

☐ No (45)

- Are bird- and vermin proof grids placed on the air inlets?

☐ Yes (53)

☐ No (7)

- Does the farm harbour vermin (rats, mice, etc.)?

☐ Much (18)

☐ Limited (6)

☐ None (36)

- Do companion animals have access to the stable(s) or the hygiene lock?

☐ Yes (10)

☐ No (50)

- Is there natural stagnant or running water within < 1 km of the farm?

☐ Yes (33)

☐ No (27)

- At what distance is the nearest turkey farm located?

☐ < 500 meter (13)

☐ 500 meter – 1 kilometer (15)

☐ > 1 kilometer (32)

- Are other animal keeping facilities located within a radius of 500 meters of your farm?

☐ No (23)

☐ Broilers (3)

☐ Laying hens (1)

☐ Veal calves (2)

☐ Dairy cattle (16)

☐ Beef cattle (7)

☐ Pigs (15)

☐ Goats (3)

- ☐ Sheep (4)
- ☐ Other, namely: (horses, geese, rabbit, turkey, poultry)

- Are there different age categories of turkeys present on the farm?

  - ☐ Yes (15)
  - ☐ No (45)

- Are there disinfection baths for vehicles present at the entry of the farm site?

  - ☐ Yes (9)
  - ☐ No (51)

- Is there a FARM-hygiene lock present?

  - ☐ Yes (14)
  - ☐ No (46)

- Are there any disinfecting footbaths present on the farm?

  - ☐ Yes (34)
  - ☐ No (26)

- Is there more than one stable present on the farm?

  - ☐ Yes (44)
  - ☐ No (16)
